# Penetrating trauma on the rise– nine-year trends of severe trauma in Sweden

**DOI:** 10.1007/s00068-024-02601-z

**Published:** 2024-07-30

**Authors:** Lina Holmberg, Kevin Mani, Fredrik Linder, Anders Wanhainen, Carl Magnus Wahlgren, Håkan Andréasson

**Affiliations:** 1 Department of Surgical Sciences, Uppsala University, Uppsala, Sweden; 2 Department of Molecular Medicine and Surgery, Karolinska Institute, Stockholm, Sweden

**Keywords:** Trauma trends, Penetrating trauma, Mortality, Intensive care unit, Emergency intervention

## Abstract

**Purpose:**

Sweden has an established trauma system involving national trauma criteria and the Swedish trauma registry (SweTrau), since over a decade. Meanwhile, the injury panorama has evolved, with an increase in gang-related violence in the Swedish community. In this study, we aimed to investigate long-term trends in mortality, management and trauma type in two major Swedish trauma centers over a nine-year period.

**Methods:**

All trauma patients with a New Injury Score (NISS) > 15 or a Trauma Alert (TA) call during 2013–2021 were identified in the participating centers’ SweTrau registries. Data were analysed regarding mortality, proportion of emergency interventions, intensive care unit (ICU) admissions, mechanism of injury and type of trauma (penetrating or blunt). To assess trends, Chi-Squared test for trend and JoinPoint regression method were used.

**Results:**

A total of 10,587 patients were included in the study. Mortality remained unchanged over time in patients with NISS > 15 (10.0-10.9%, *p* = 0.963) but increased in patients with a TA *and* NISS < 15 (1.3-2.7%, *p* = 0.005). For NISS > 15, the proportion undergoing emergency interventions was stable (53.9%-48.8%, *p* = 0.297) while ICU admissions declined (62.1%-45.7%, *p* < 0.001). Penetrating trauma increased (12.4-19.6%, *p* < 0.001), including knife (10.0-15.7%, *p* < 0.001) and gunshot wounds (2.3-3.8%, *p* < 0.001), whereas accidents involving motorcycles (8.8%-7.0%, *p* = 0.004) and pedestrians (5.3%-2.2%, *p* < 0.001) decreased. Assaults (both penetrating and blunt) increased from 14.7 to 21.4% (*p* < 0.001).

**Conclusions:**

In this trend analysis at two major Swedish trauma centers during 2013–2021, penetrating trauma increased with over 50% while traffic injuries decreased. The rise in mortality in patients with a TA *and* NISS < 15 is concerning and requires further evaluation, as do the reduction in ICU admissions.

**Supplementary Information:**

The online version contains supplementary material available at 10.1007/s00068-024-02601-z.

## Introduction

Trauma is affecting all ages throughout the population and is a significant burden to society. The occurrence of trauma in a population is affected by societal changes, and such changes may also affect the management algorithm for trauma as well as trauma outcome. In Sweden, as in most European countries, blunt trauma is the dominating injury type, and knife injuries are more common than gunshot wounds among patients with penetrating trauma. Nonetheless, over the past decades in Sweden, there has been a 2-fold increase in deadly violence due to firearms [1]. During the years 2000–2019, Sweden has climbed from the bottom to the top of gunshot wound homicides in Europe, with the absolute majority of cases being gang-related and linked to illicit drugs, illegal weapons and socially disadvantaged areas [2]. This dramatic (and in Europe previously unseen) increase takes off steeply from 2013 and onward, with Sweden leading the list since 2018. In 2021, Sweden had 4 deaths per million inhabitants due to firearm violence, compared to an average of 1.6 deaths per million inhabitants in Europe [2]. The most notable increase is seen in the age group 20–29 years old, with 18 deaths per million inhabitants in Sweden, compared to 0–4 deaths per million in most other European countries [2]. This worrisome development calls for an urgent need to identify where the most potential of improvement in trauma care lies, by mapping changes and trends in the trauma panorama. In order to safely modify the way to prepare for, treat and prevent trauma, it is important to have reliable data and research to base such recommendations on. In Sweden, a uniform system for trauma call activation exists; the Swedish National Trauma Triage Criteria (SNTTC) [3], with two levels of trauma call: Trauma Alert (TA, highest) and Trauma Response (TR, lowest). Furthermore, there is a national trauma registry, the Swedish trauma registry (SweTrau), implemented in 2011 [4] and recently validated [5]. All these parts of the Swedish trauma system make up a solid foundation for studying trends of trauma.

Objectives.

The current paper aims to assess changes in trauma care and trauma patterns in Sweden over a nine-year period, using data from SweTrau at two major trauma centers. The primary study question was how the outcome of trauma care, defined as 30-day mortality after severe trauma, has changed over time in patients with: New Injury Score (NISS) > 15, a TA-call *and* NISS < 15, penetrating and blunt injuries. Secondary study questions included analysis of the proportion of patients with severe trauma and an emergency intervention or an admission to the intensive care unit (ICU), and changes in the patterns of mechanisms of injury (including penetrating vs. blunt trauma).

## Material & methods

### Study design: Setting and population

All trauma patients with NISS > 15 or a TA (regardless of NISS) from two Swedish trauma centers (Karolinska university hospital in Stockholm and Uppsala university hospital in Uppsala) between the years 2013 to 2021 were examined. These trauma units were the only hospitals with complete registration in SweTrau throughout the study period and therefore selected for the study, in order to minimise bias due to missing data. They serve a primary catchment area of 2.8 million inhabitants in central Sweden, comprising about 27% of the total Swedish population. Both centers offer comprehensive trauma care including all trauma subspecialities, and have a tertiary care status for treatment of complex trauma patients within their respective regions (total population catchment area approximately 4.6 million). The selection of inclusion criteria was to ensure that all severely injured trauma patients would be captured, so that for example patients with penetrating trauma that did not reach NISS > 15 but still required an emergency intervention were not missed. The choice of NISS over Injury Severity Score (ISS) [6] was made on the basis of NISS being an inclusion criteria of SweTrau, as well as being better at describing penetrating injuries [7], in-hospital mortality [8] and post-trauma multiorgan failure [9]. The population was divided into groups (TA *and* NISS < 15, NISS > 15, penetrating and blunt trauma) and the annual percentage of the different outcomes (mortality, emergency intervention, ICU-admission) as well as the percentage of each mechanism of Injury (MOI) was calculated and trends assessed. We did not use incidence rates since several hospitals in Stockholm received severely injured trauma patients during the study period, making the population denominator too uncertain. However, an additional analysis of incidence rate regarding severe trauma (non-referrals) in the primary catchment area was made, as an approximate estimation to exclude a significant change in either population or trauma numbers which might affect the results. Finally, the trend of violence (both penetrating and blunt) was investigated in a subanalysis.

### Data sources and variables

The time period of the study was selected to include data from the complete inception of the SweTrau registry (initiation phase in 2011 to 2012 with fully established registry from 2013). SweTrau includes all trauma patients with a trauma call (either a TA or TR) or a NISS > 15. The variables of SweTrau are based on the internationally acknowledged consensus document “The Utstein Template of Trauma” [10, 11] and contains patient characteristics, such as social security number, age, sex and American Society of Anesthesiologists (ASA) physical status score, as well as pre-hospital and in-hospital data on injuries and performed emergency interventions. These are defined as thoracotomy, laparotomy, pelvic packing, revascularization, endovascular intervention, craniotomy, intracranial pressure measurement or other (chest tube, extern fixation of fracture, major surgery of fractures or wound revision in the operating room). The registry also records days on ventilator in the ICU, discharge destination (home, rehabilitation, other care facility, morgue etc.) and injury type: penetrating (e.g. penetrates through the tissue) or blunt (injury resulting from a person being hit by or hitting another object). The injury type should not be confused with the mechanism of injury; a patient may have a blunt mechanism of injury but still sustain a penetrating injury. The 30-day mortality variable is verified during the registration in SweTrau via each hospitals electronic records which are linked to the Swedish Tax Agency that files all death certificates in Sweden, thus ensuring 100% accuracy for Swedish citizens.

### Statistical analyses

Statistical analyses were performed with IBM SPSS Statistics, version 28.0.1.0 (IBM Corp., Armonk, N.Y., USA), JoinPoint Regression Program, version 5.0.2 (Statistical Methodology and Applications Branch, Surveillance Research Program, National Cancer Institute) and with Microsoft Excel for Mac, version 16.74. Medcalc.org was used to obtain the confidence intervals for the incidence rate in Supplemental material 1. Categorical data were analyzed with Chi-Squared test and numerical data with Mann-Whitney U test (two groups) or Kruskal-Wallis test (more than two groups: comparing the distribution of NISS between different years). Trends were analyzed with Chi-Squared test for trend and JoinPoint regression model (also called segmental regression) which uses joinpoints (changing points) to fit the best model and test if the trend is statistically significant. This results in an annual average percent change (AAPC) which is a more intuitive measurement to interpret. The level of significance was set at a p-value < 0.05.

## Results

### Population descriptives

**Fig. 1:**
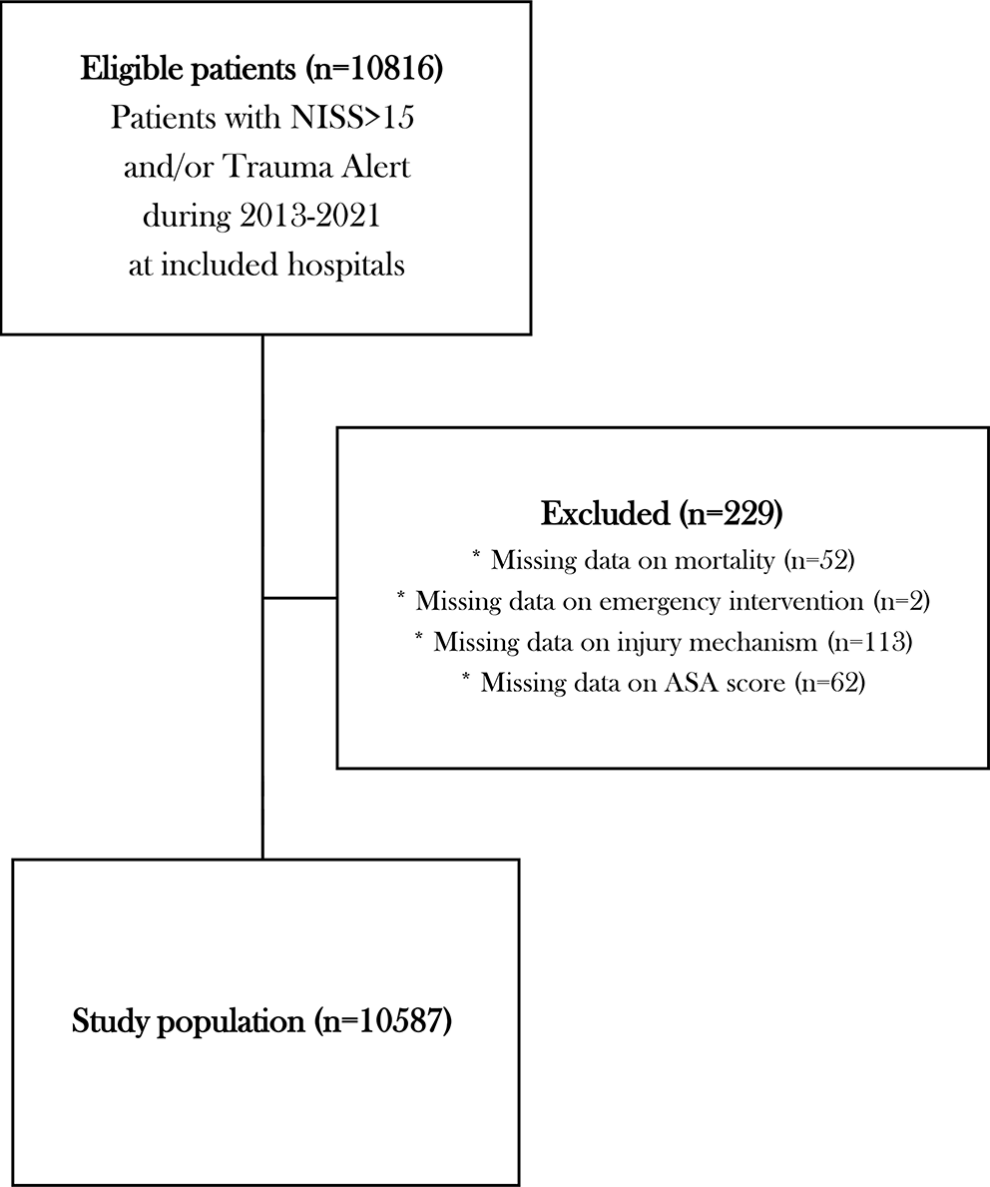
Flowchart of patient population. NISS = New Injury Severity Score. ASA score = American Society of Anesthesiologists physical status score

The eligible number of patients was 10,816 which, after exclusion due to missing data, led to a study population of 10,587 patients (Fig. 1). Patient characteristics of the population, as well as for the groups NISS > 15, TA *and* NISS < 15, penetrating trauma and blunt trauma is presented in Table 1. The group of penetrating trauma was younger and had a higher proportion of males and less comorbidities, as well as a lower NISS score, than the group of blunt trauma. Patients with TA *and* NISS < 15 experienced more penetrating trauma than patients with NISS > 15, while also being younger and having a lower ASA and NISS score.

**Table 1:** Characteristics. NISS = New Injury Severity Score. IQR = interquartile range. ASA score = American Society of Anesthesiologists physical status score. ^1^Chi-Squared test. ^2^Mann-Whitney U test. * = *p* < 0.05

Characteristics	Population (*n* = 10,587)		NISS > 15 (*n* = 5724)	TA and NISS < 15 (*n* = 4863)	*p*-value	Penetrating trauma (*n* = 1796)	Blunt trauma (*n* = 8791)	*p*-value
Male sex, n (%)	7752 (73.2)		4235 (74.0)	3517 (72.3)	0.054^1^	1619 (90.1)	6133 (69.8)	< 0.001*^1^
Age– years, median (IQR)	43 (26–61)		49 (29–66)	36 (24–53)	0.000*^2^	29 (22–42)	46 (28–64)	0.000*^2^
Age > 65 years, n (%)	2234 (21.1)		1562 (27.3)	672 (13.8)	< 0.001*^1^	95 (5.3)	2139 (24.3)	< 0.001*^1^
Children < 15 years old, n (%)	159 (1.5)		131 (2.3)	28 (0.6)	< 0.001*^1^	10 (0.6)	149 (1.7)	< 0.001*^1^
ASA score 3 or higher, n (%)	1911 (18.1)		1288 (22.5)	623 (12.8)	< 0.001*^1^	164 (9.1)	1747 (19.9)	< 0.001*^1^
NISS– median (IQR)	17 (5–27)		27 (19–34)	5 (2–9)	0.000*^2^	9 (2–19)	17 (6–27)	0.000*^2^
Penetrating trauma, n (%)	1796 (17.0)		566 (9.9)	1230 (25.3)	< 0.001*^1^	1796 (100.0)	0 (0.0)	NA
30-day mortality, n (%)	828 (7.8)		744 (13.0)	84 (1.7)	< 0.001*^1^	131 (7.3)	697 (7.9)	0.361^1^

### Main results and outcome data

#### Mortality

**Fig. 2:**
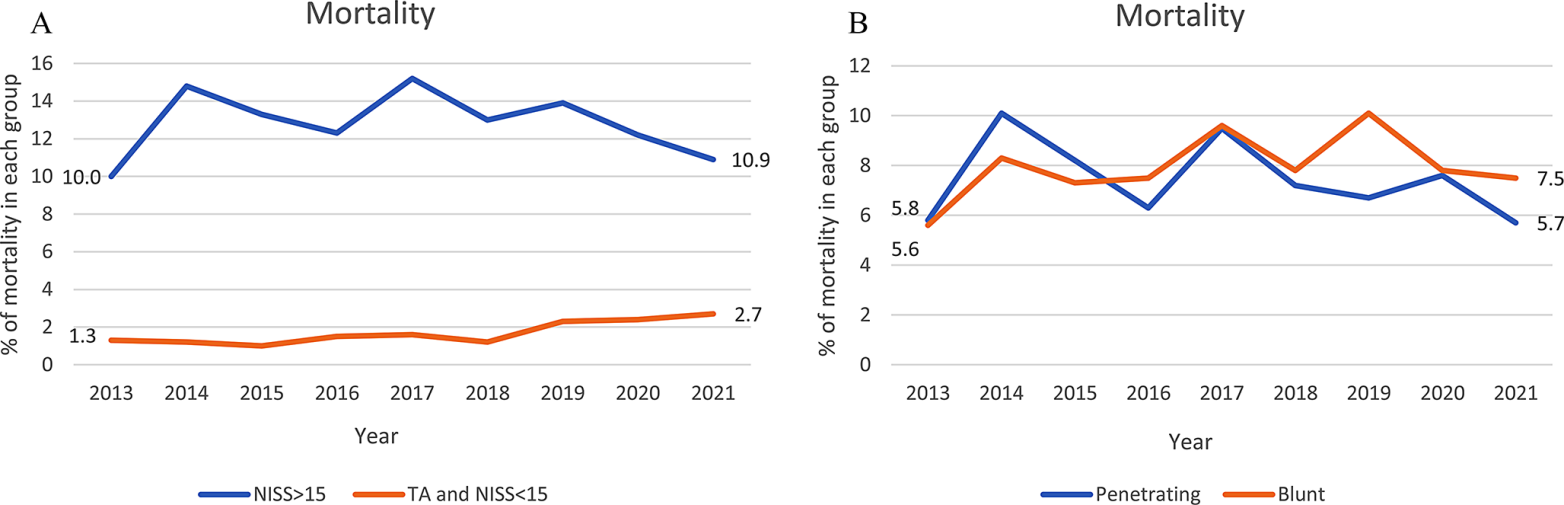
**a&b** Trends in mortality for the groups TA and NISS>15 vs. NISS<15 and penetrating trauma vs. blunt trauma

The overall 30-day mortality in the population was 7.8% with a significantly higher mortality for patients with NISS > 15 than patients with a TA *and* NISS < 15 (13.0% vs. 1.7%, *p* < 0.001, Table 1). The mortality remained unchanged over time for the groups penetrating trauma, blunt trauma and NISS > 15, while it increased for patients with TA *and* NISS < 15 (1.3–2.7%, *p* = 0.005) (Table 2; Fig. 2). The majority of deaths in the group of TA *and* NISS < 15 (52.4%, 44 of 84 patients) occurred during the last third of the study period, between 2019 and 2021 (data not shown). Among these 44 patients there were seven deaths from knife injuries and gunshot wounds (GSW) (compared to only two during the first six year of the study), and suicide from blunt trauma accounted for five deaths during this period, as opposed to one during 2013–2018. Furthermore, although there were no deaths in the emergency department (ED) in the first six years of the study, thirteen patients with a TA *and* NISS < 15 died in the ED during 2019–2021 (data not shown). Eleven of these patientshad a pre-hospital cardiac arrest, comprising one young man with a GSW and ten patients categorized as blunt trauma, aged between 51 and 67 years, with a NISS of 1–3 (eight patients), 8 (one patient) and 14 (one patient). When performing a subanalysis (data not shown) on the mortality of TA *and* NISS < 15, there was a significant increasing mortality trend (*p* = 0.002) in blunt trauma (1.3 − 3.1%, a total of 75 deceased patients). The mortality trend for penetrating trauma was not significant (*p* = 0.311) and varied from 0 to 2.1% (in 2021) with a total of nine deceased patients.

**Table 2:** Chi-squared test for trend and annual average percentage change. AAPC = annual average percentage change. CI = confidence interval. NISS = New Injury Severity Score. ICU = intensive care unit. MOI = mechanism of injury. GSW = gunshot wound. MCC = motorcycle crash, MVC = motor vehicle crash, other vehicle (e.g. ship, airplane, train, tram), other (e.g. burns). NA = not applicable, too few patients. NC = not calculated due to not significant result with x^2^ for trend (both methods need to be significant to conclude a trend). * = *p* < 0.05

	Chi-Squared test for trend *p*-value	JoinPoint regression AAPC (CI)
**Mortality**		
*NISS > 15*	0.963	-0.75 (-5.17, 4.11)
*TA and NISS < 15*	0.005*	12.24* (7.53, 18.36)
*Penetrating trauma*	0.465	-2.90 (-10.85, 7.33)
*Blunt trauma*	0.079	1.14 (-3.95, 8.82)
**Emergency Intervention**		
*NISS > 15*	0.297	-0.64 (-2.34, 1.15)
*TA and NISS < 15*	0.029*	1.51 (-0.73, 4.21)
*Penetrating trauma*	0.221	0.01 (-2.22, 2.49)
*Blunt trauma*	0.909	-0.11 (-1.66, 1.51)
**ICU**		
*NISS > 15*	< 0.001*	-3.00* (-4.61, -1.38)
*TA and NISS < 15*	0.993	-0.57 (-5.48, 5.50)
*Penetrating trauma*	0.001*	-7.23* (-8.47, -5.90)
*Blunt trauma*	0.030*	-1.93* (-3.22, -0.59)
**Increasing MOI**		
*Knife*	< 0.001*	7.46* (4.82, 10.81)
*Hit by blunt object*	0.006*	3.32* (1.54, 5.35)
*GSW*	< 0.001*	7.12* (4.82, 9.97)
**Decreasing MOI**		
*MCC*	0.004*	-3.57* (-5.89, -1.26)
*Pedestrian*	< 0.001*	-8.84* (-15.14, -2.80)
**Unchanged MOI**		
*MVC*	< 0.001*	-3.14 (-7.77, 1,56)
*Bicycle*	0.049*	2.55 (-1.21, 6.98)
*Other vehicle*	0.104	NC
*Low fall*	0.851	NC
*High fall*	0.028*	-1.51 (-3.11, 0.16)
*Explosion*	NA	NA
*Other*	0.398	NC

#### Emergency intervention

**Fig. 3:**
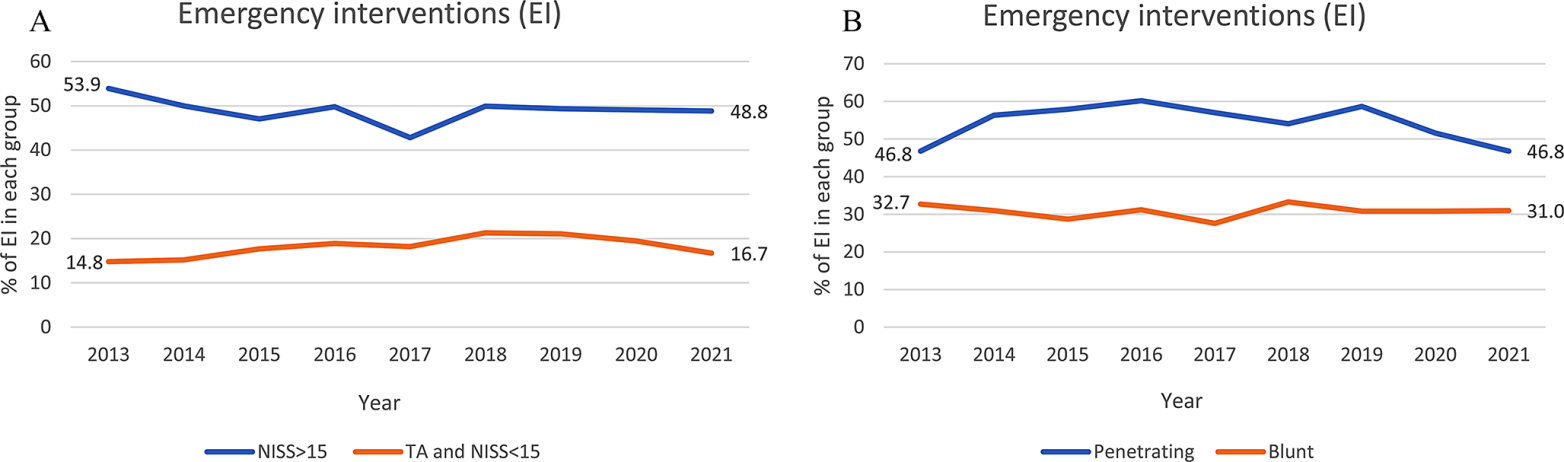
**a&b** Trends in emergency intervention for the groups NISS>15 vs. TA and NISS<15 and penetrating trauma vs. blunt trauma

The rate of emergency interventions for NISS > 15 remained around 50% (Fig. 3a). The four most common emergency interventions in the groups NISS > 15 and blunt trauma were the same: craniotomy, major surgery of fractures, chest tube and intracranial pressure measurement (data not shown). Patients with penetrating trauma and a TA *and* NISS < 15 shared three out of the four most common emergency interventions: wound revision in the operating room, laparotomy and chest tube, with thoracotomy being in the top four with penetrating trauma as opposed to major surgery of fractures in patients with a TA *and* NISS < 15 (data not shown). Overall, there were no significant trends in the rate of emergency interventions for patients with NISS > 15, nor for patients with penetrating or blunt trauma (Table 2). For patients with a TA *and* NISS < 15, the two statistical methods had divergent findings, with Chi-Squared test for trend suggesting a significant change in emergency interventions rate over time (14.8–16.7%, *p* = 0.029), whilst this change was not significant when assessed with JoinPoint regression (AAPC 1.51; CI -0.73, 4.21, Table 2). The graphic changes in the rate of emergency interventions per year are visualized in Fig. 2.

#### Intensive care unit admission

**Fig. 4:**
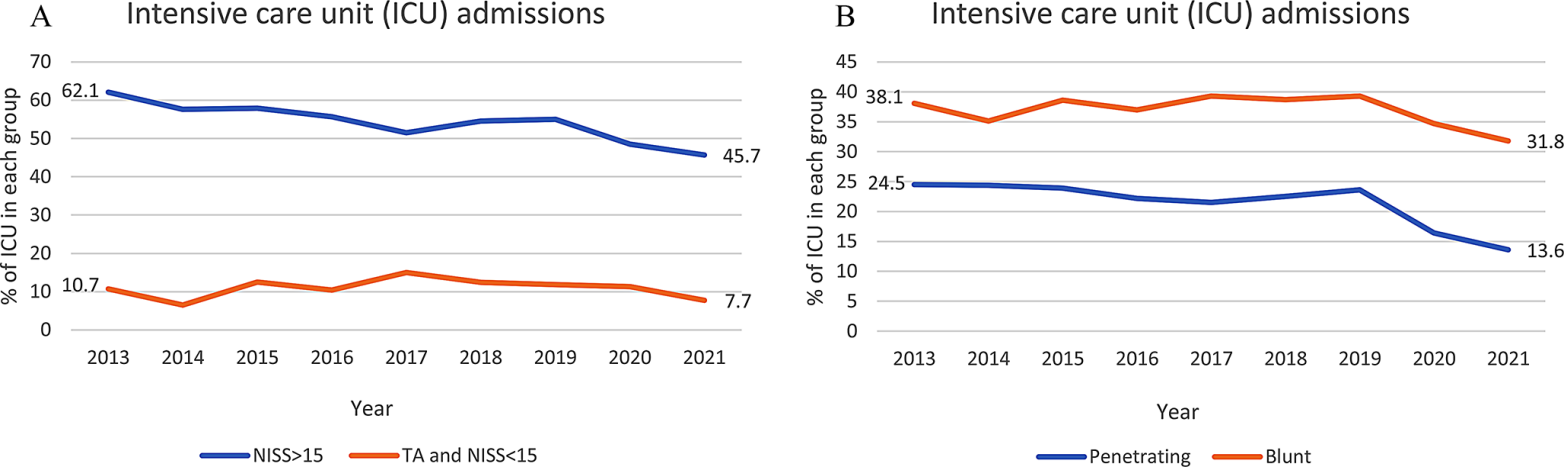
**a&b** Trends in intensive care unit admissions for the groups NISS>15 vs. TA and NISS<15 and penetrating trauma vs. blunt trauma

Figure 4 shows the rate of admission to ICU after trauma. Admissions to the ICU decreased for patients with NISS > 15 (62.1–45.7%, *p* < 0.001), whilst it was stable for patients with a TA *and* NISS < 15 (Table 2). When assessing rate of ICU admission for blunt and penetrating trauma, a clear decrease in admissions in both groups was observed from 2020 and onwards (Fig. 4b; Table 2).

#### Mechanisms of injury

**Fig. 5:**
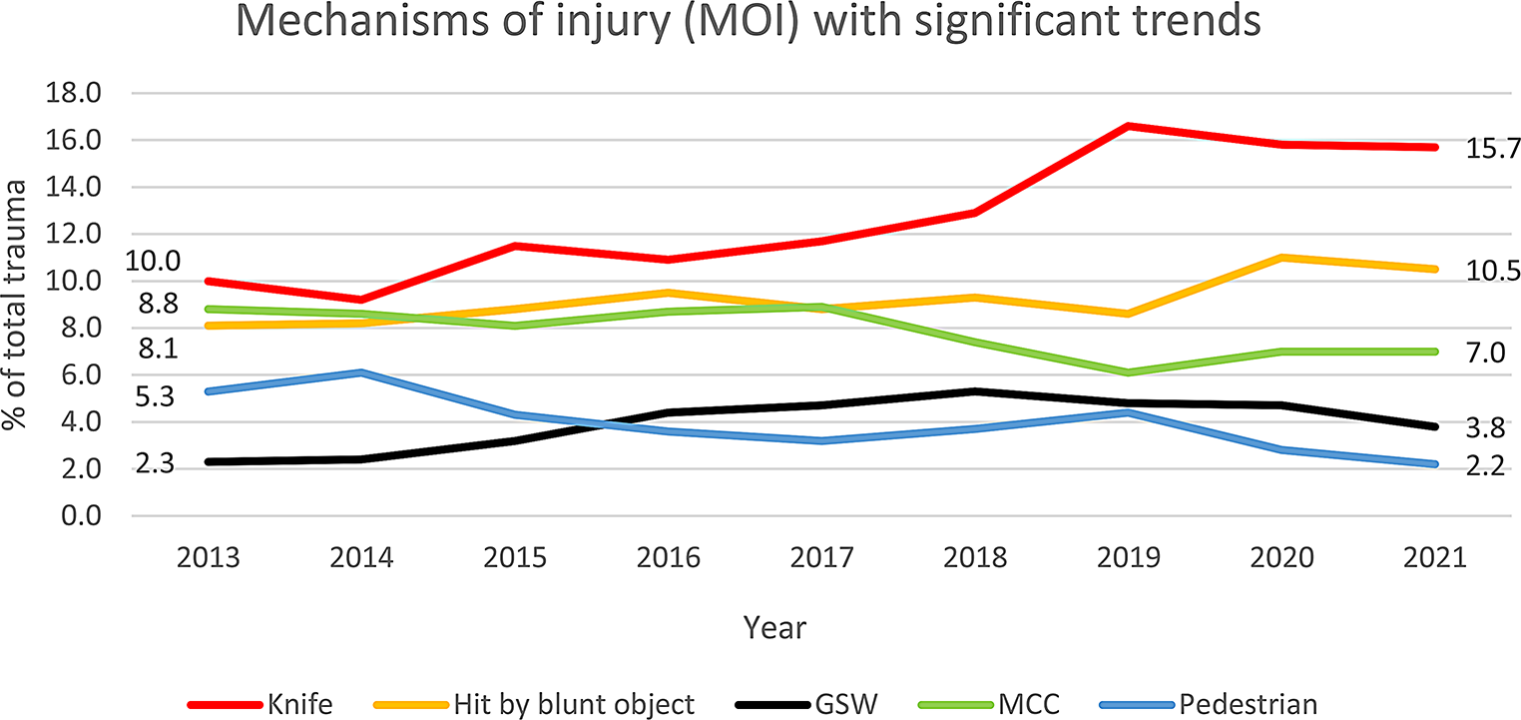
Mechanisms of injury (MOI) with significant trends. Knife (e.g. knife, sword, other sharp object), Hit by blunt object (e.g. tree, branch, brick, pole etc.), GSW = gunshot wound, MCC = motorcycle crash, Pedestrian (e.g. traffic accident involving pedestrian, wheelchair, inlines, skateboard etc.). The following MOI:s did not exhibit significant trends over the study period and are therefore not shown: MVC (motor vehicle crash), Bicycle accident, Other vehicle (e.g. ship, airplane, train, tram), Low fall, High fall, Explosion, Other (e.g. burns)

The rate of mechanisms of injury per year are presented in Fig. 5. This figure focuses on mechanisms of injury with a significant change over time when analyzed with both Chi-Squared test for trend and JoinPoint regression (Table 2). Assaults with knife (10.0–15.7%, *p* < 0.001), GSW (2.3–3.8%, *p* < 0.001) and hit by blunt object (8.1–10.5%, *p* = 0.006) increased, while traffic accidents involving motorcycles (MCC) (8.8–7.0%, *p* = 0.004) and pedestrians (5.3–2.2%, *p* < 0.001) decreased. The rest of the mechanisms of injury, for example falls and motor vehicle crashes, did not exhibit significant trends when examined with both methods (Table 2).

### Additional analyses

#### Penetrating trauma, gender proportion and NISS

Table 3 displays trends in penetrating and blunt trauma. The proportion of patients subjected to penetrating trauma increased from 12.4 to 19.6% (*p* < 0.001). There was no change in laparotomy or thoracotomy rates among those with penetrating trauma. The fraction of male patients remained stable at approximately 90% for penetrating trauma and 70% for blunt trauma, while a median of NISS 13 was maintained for penetrating trauma and 17 for blunt trauma, although there were some fluctuations for penetrating trauma (Table 3).

**Table 3:** Incidence of blunt and penetrating trauma. IQR = interquartile range. ^1^Chi-Squared test for trend. ^2^Kruskal-Wallis test. * = *p* < 0.05

Severe trauma (*n* = 10,587)	2013 (*n* = 1122)	2014 (*n* = 1060)	2015 (*n* = 1060)	2016 (*n* = 1182)	2017 (*n* = 1172)	2018 (*n* = 1132)	2019 (*n* = 1172)	2020 (*n* = 1335)	2021 (*n* = 1352)	*p*-value
**Blunt**,** n (%)**	983 (87.6)	941 (88.8)	901 (85.0)	1006 (85.1)	972 (82.9)	923 (81.5)	918 (78.3)	1060 (79.4)	1087 (80.4)	< 0.001*^1^
*Male sex*,* n (%)*	*705 (71.7)*	*658 (69.9)*	*630 (69.9)*	*707 (70.3)*	*680 (70.0)*	*641 (69.4)*	*635 (69.2)*	*729 (68.8)*	*748 (68.8)*	0.118^1^
*NISS median (IQR)*	*17 (6–27)*	*17 (6–29)*	*17 (6–27)*	*17 (6–27)*	*17 (9–27)*	*17 (6–27)*	*17 (6–27)*	*17 (6–27)*	*17 (6–27)*	0.188^2^
**Penetrating n (%)**	139 (12.4)	119 (11.2)	159 (15.0)	176 (14.9)	200 (17.1)	209 (18.5)	254 (21.7)	275 (20.6)	265 (19.6)	< 0.001*
*Male sex*,* n (%)*	*130 (93.5)*	*108 (90.8)*	*139 (87.4)*	*165 (93.8)*	*184 (92.0)*	*188 (90.0)*	*223 (87.8)*	*250 (90.9)*	*232 (87.5)*	0.099^1^
*NISS median (IQR)*	*13 (2–17)*	*17 (3–22)*	*14 (3–18)*	*15 (2–22)*	*16 (3–22)*	*13 (2–18)*	*13 (3–19)*	*13 (3–18)*	*13 (2–18)*	0.024*^2^
*Laparotomy n (%)*	*7 (5.0)*	*7 (5.9)*	*7 (4.4)*	*9 (5.1)*	*9 (4.5)*	*8 (3.8)*	*8 (3.1)*	*10 (3.6)*	*8 (3.0)*	0.099^1^
*Thoracotomy n (%)*	*16 (11.5)*	*23 (19.3)*	*19 (11.9)*	*15 (8.5)*	*17 (8.5)*	*22 (10.5)*	*34 (13.4)*	*32 (11.6)*	*38 (14.3)*	0.810^1^

#### Incidence of severe trauma

There was no significant trend in the incidence of severe trauma patients (non-referrals) per year at the included hospitals. The incidence varied from 34 to 39 per 100 000 inhabitants per year during the study period with overlapping 95% confidence intervals, as seen in Supplemental material 1.

#### Violence trends

Assaults increased significantly during the study period, from 14.7 to 21.4% (*p* < 0.001) as well as the percentage of assaults combined with self-harm and suicides (21.8 − 28.5%, Supplemental material 2).

## Discussion

### Key results

This study, examining 10,587 patients for a time period of nearly a decade, is to our knowledge the largest study in Sweden reporting trends in trauma. During the study period, there was an increase in mortality in trauma patients with a TA and NISS < 15, no change in the rate of emergency interventions and a decline in ICU admissions in all groups except TA *and* NISS < 15. Penetrating trauma increased significantly from 12.4 to 19.6%, and there was an increase in assaults (both penetrating and blunt) from 14.7 to 21.4%.

### Interpretation

#### Mortality

Overall mortality in the group of NISS > 15 was 13.0%– less than in studies from Denmark [12], the USA [13] and Japan [14] but comparable to a previous Swedish study [3]. The mortality also compares very well when looking at SweTrau’s yearly reports, were the mortality in the group NISS > 15 between the years 2013–2021 varies from 14 to 20%, with a mean of around 17%. However, the mortality in SweTrau reflects a mixture of university hospitals and regional hospitals, in contrast to our study’s two major university trauma centers, which could possibly explain the higher mortality rate in SweTrau. Further, our results are similar to an Australian study that showed no significant changes in mortality during the years 2006–2016 [15], with one worrisome exception in our study; the group with a TA *and* NISS < 15 where mortality has risen from 1.3 to 2.7%. One could hypothesize that the implementation of the SNTTC in 2017 could have led to a more stringent use of the highest trauma call (TA), possibly resulting in a change of presentation of injured patients with a TA *and* NISS < 15 to ED: s and thus explaining part of this observed increase in mortality among NISS < 15 patients. However, in a Swedish study from 2019 [16], it was shown that both the number of TA: s and the proportion of patients with ISS < 15 among TA: s were the same before and after the implementation of the new criteria, which do not support this idea. The increase in mortality for patients with a TA *and* NISS < 15 is visible from 2019 and onward and an important proportion was related to elderly patients with blunt trauma and a low NISS that died in the ED. The low NISS (1–3) in these patients is surprising since it does not necessarily explain a traumatic cardiac arrest from blunt trauma, but could rather be due to e.g. rib fractures caused by cardiopulmonary resuscitation. Taking all this into account, including patient age, one must consider the possibility of these patients having been mis-triaged as trauma patients whilst instead suffering a non-traumatic cardiac arrest, subsequently causing the patient’s motor vehicle crash or fall. A factor that somewhat contradicts this, however, is the quality improvement variable “morbidity & mortality conferences” (M&M) in SweTrau. All these patients have been discussed at M&M:s to determine if the patient is a trauma patient or not, and normally cases that are not primary trauma would have been excluded from the registry after such discussion. Moreover, the subanalysis of the mortality in TA *and* NISS < 15 displayed a significant increase in blunt trauma only. However, there were only nine deaths from penetrating trauma (of which seven occurred 2019–2021), why there may be a risk of a type II-error. A larger population might have shown a mortality increase in penetrating trauma as well. On closer examination of the TA *and* NISS < 15 group, there was a striking difference in the number of deaths from knife injuries, GSW and suicides from blunt trauma during the study period, resulting in twelve fatalities during the last three years (2019–2021) compared to only three during the first six years (2013–2018). In summary, deaths from penetrating trauma and suicides, together with the blunt trauma patients who died in the ED, constituted more than half of the deceased patients with a TA *and* NISS < 15 during 2019–2021, coinciding with the increase in mortality in this group. The change in the trauma panorama in Sweden, with a clear increase of both penetrating and blunt assaults, thus can have contributed to this outcome.

#### Emergency intervention & intensive care unit admissions

Emergency interventions did not change during the study period, underlined by the fact that laparotomy and thoracotomy in penetrating trauma showed no significant trends. The overall decrease in ICU admissions is however of interest, with an even sharper decline during the COVID-19 pandemic years of 2020–2021. This does not correspond to findings from other international studies [17, 18], where the ICU proportions of patients requiring ICU admission was unchanged during COVID-19. Furthermore, the number of trauma patients in our study actually increased during the years 2020–2021, unlike many other countries that practiced lockdown [17–20], and the increase in severe trauma cannot be explained in its entirety by a rise in population. This could be interpreted as a very concerning finding, indicating that trauma patients in Sweden were down-prioritized from the ICU during COVID-19. Nonetheless, as earlier discussed there was no increase in mortality during the study period except for the group with a TA *and* NISS < 15 and, on closer examination, there was no decrease in the proportions of ICU admissions in deceased patients in this group. This indicates that even if fewer trauma patients were indeed admitted to the ICU, patient safety was still maintained. The current data therefore suggests that a larger proportion of severe trauma could be managed at a somewhat lower care level with the same result.

#### Mechanisms of injury

During the study period, we found that “being hit by a blunt object” increased as a mechanism of injury, together with other assaults (knife-inflicted trauma and GSW)– in contrast to the findings of a study from the USA between 2005 and 2014 [21] but consistent with a Danish study between 2010 and 2019 [12]. We also found that traffic injuries such as MCC and pedestrian accidents decreased, in line with other studies [12, 14, 21]. These changes could be interpreted as a shift towards violence being a more prevalent cause of injury while traffic related trauma decreases, indicating areas to focus on when discussing trauma prevention. Of note, we could not see a significant change in fall injuries, contrary to other studies that have reported a trend of higher incidence of falls [12, 14, 21, 22]. One possible explanation to this could be that this study only examines severe trauma, not the whole trauma population.

#### Penetrating trauma

The proportion of penetrating trauma increased with more than 50% during the study period (12.4–19.6%, *p* < 0.001), but the mortality remained at just below 6% with some variations over the years. The increase in penetrating trauma in Sweden is supported by other studies [23, 24], and one has to remember that patients that are pronounced dead at scene are not included in SweTrau. This can lead to false low mortality numbers, as showed in a Swedish study [23] where death due to violence was increased but not in-hospital mortality for penetrating trauma.

### Strengths, limitations and generalisability

The major strengths of our study are the substantial size of the population and the extensive time period examined, as well as the inclusion of two out of in total seven university hospitals in Sweden. Using two different statistical methods (JoinPoint regression [25] and Chi-Squared test for trend) also strengthens the validity of the results. JoinPoint is a common way to describe trends in incidence [21, 26] and includes the advantage of a measurement (AAPC) that is easy to comprehend and compare. The additional analysis of estimated incidence of severe trauma in the primary catchment area displayed no significant trend during the study, reinforcing the validity of the percentage rates for the different outcomes. The retrospective nature of this paper carries the usual limitations of a registry based study, which makes more in-depth analyses of individual cases difficult. Also, any international comparisons of the results should be done with the Swedish context of our study in mind. A possible bias is the excluded patients; however, these were scarce (about 2%) and the potential impact should therefore be minimal. Finally, since patients that die on the scene of trauma are not included in SweTrau, the mortality rate needs to be interpreted considering this, and our study therefore only reflects the in-hospital mortality.

### Implications and future perspectives

This article has highlighted a very worrisome increase in penetrating trauma and assaults, concerning not only the healthcare sector but the society as a whole. The fact that on-scene mortality is not recorded only underlines the need of further studies combining these parameters as well as mapping other relevant epidemiological aspects. The study finding of lower rate of ICU utilization during the study period, with maintained low mortality in this group of trauma patients, raises the possibility that a lower care level may be appropriate in selected trauma cases, which could improve resource utilisation and access to ICU. Further studies comparing patients treated at a dedicated trauma ward or intermediate ward with patients treated at ICU are needed.

## Conclusion

Penetrating trauma with knife injuries and GSW increased with more than 50% at two major Swedish trauma centers during 2013–2021, with decreasing traffic related injuries such as MCC and pedestrian accidents. Assaults also increased from 14.7 to 21.4%, while no trend was seen in the proportion of emergency interventions performed. Patients with a TA and NISS < 15 displayed a troublesome finding of a more than a doubled mortality, primarily during 2019–2021, which clearly merits further investigation. Additionally, a clear decrease in ICU admissions– not correlated with the increased mortality in the TA and NISS < 15 group– was noted, indicating that a lower care level may be safe for a larger proportion of trauma patients.

## Electronic supplementary material

Below is the link to the electronic supplementary material.

Supplementary Material 1

## Data Availability

Data available from the authors upon reasonable request.
