# Supplementary material for: Penetrating trauma on the rise– nine-year trends of severe trauma in Sweden
Source: Eur J Trauma Emerg Surg. 2024 Jul 30;50(6):3189–97. doi: 10.1007/s00068-024-02601-z (PMC11666756; doi:10.1007/s00068-024-02601-z)
Supplement: Supplementary file 1 — Supplementary Material 1 [file 68_2024_2601_MOESM1_ESM.docx]

| **Year** | 2013 | 2014 | 2015 | 2016 | 2017 | 2018 | 2019 | 2020 | 2021 |
| --- | --- | --- | --- | --- | --- | --- | --- | --- | --- |
| **Primary catchment area, n** | 2 508 523 | 2 546 986 | 2 585 603 | 2 630 433 | 2 677 114 | 2 720 478 | 2 760 794 | 2 780 384 | 2 810 165 |
| **Severe trauma (no referrals), n** | 961 | 903 | 905 | 996 | 982 | 936 | 947 | 1067 | 1101 |
| **Incidence rate per 100000 (95% CI)** | 38 (36-41) | 35 (33-38) | 35 (33-37) | 38 (36-40) | 37 (34-39) | 34 (32-37) | 34 (32-37) | 38 (36-41) | 39 (37-42) |

Supplemental material 1 – Incidence rate of severe trauma (except referral patients) in the primary catchment area.

Source: Statistics Sweden, www.scb.se.
